# *Cordyceps militaris* extracts and cordycepin ameliorate type 2 diabetes mellitus by modulating the gut microbiota and metabolites

**DOI:** 10.3389/fphar.2023.1134429

**Published:** 2023-03-09

**Authors:** Xinyuan Liu, Mengqian Dun, Tongtong Jian, Yuqing Sun, Mingyu Wang, Guoying Zhang, Jianya Ling

**Affiliations:** ^1^ State Key Laboratory of Microbial Technology, Shandong University, Qingdao, SD, China; ^2^ Shandong University of Traditional Chinese Medicine, Jinan, SD, China

**Keywords:** T2DM, C. militaris, gut microbiota, 16S rDNA, metabolomics, pathway

## Abstract

**Introduction:** *Cordyceps militaris*, which has many potential medicinal properties, has rarely been reported to alleviate type 2 diabetes mellitus (T2DM).

**Methods:** The effects of *C. militaris* extracts (CE) and cordycepin (CCS) on high-fat diet and streptozotocin (STZ) induced T2DM mice were analysed by gut microbiome and metabolomics methods in this study.

**Results:** The results demonstrated that glucose and lipid metabolism parameters, oxidative stress biomarkers and inflammation cytokines were down-regulated in the CCS and CE groups. A comparative analysis of the fecal samples from mice in the model and experimental groups showed that experimental groups resulted in a higher abundance of Firmicutes/Bacteroidetes.

**Conclusion:** This study provides evidence that *C. militaris* can be used as a food supplement to relieve T2DM, which provides a promising prospect for new functional food in it.

## Highlights

(1) Based on the 16S rDNA gene sequencing and metabolomics analyses, the underlying mechanism of cordycepin (CCS) and *C. militaris* extracts (CE) on the amelioration of T2DM by modulating the gut microbiota and metabolites was firstly investigated
(2) CCS and CE showed beneficial effect on modulating gut microbiota composition and structure.
(3) CCS and CE presented beneficial effects on modulating metabolites, including phenols, fatty acids, phenylacetones, pyrimidine nucleosides, carboxylic acids, etc
(4) Further analyses of metabolic pathways indicated that the therapeutic effect of CCS and CE might be related predominantly to PI3K/Akt/mTOR pathway.

## 1 Introduction

Diabetes mellitus (DM) is a metabolic disease that exists widely in the world, and its clinical manifestation is that long-term blood sugar is higher than the standard value (Maxwell, K. G. et al., 2020). Diabetes and its complications can cause damage to the eyes, kidneys, nerves, heart, blood vessels and other tissues and organs of patients, and even lead to death. As of 2021, 537 million adults worldwide have diabetes, and about 6.7 million people have died from the disease. T2DM is a common type of diabetes, and it is the most persistent and common metabolic disease as a global public health issue. The pathogenesis of T2DM is considered as impaired insulin secretion and decreased insulin sensitivity, about 90% of diabetic patients are diagnosed with T2DM (Hameed I et al., 2015). Mechanisms of new drugs for treating T2DM include targeting *ß* cells and the incretin axis. Traditional hypoglycemic drugs can only be used to control the level of blood glucose to suppress complications such as metformin, glibenclamide and miglitol (Chiriacò M et al., 2019). For centuries, edible fungi and natural plants have attracted extensive attention and been used for pharmacological research due to their low toxicity and beneficial to human body. Some edible fungi, such as *Ganoderma lucidum*, *Grifola frondose*, *Hericium erinaceus*, *Phellinus linteus*, *Auricularia auricular*, *C. militaris* have shown potential anti-diabetic effects (Chen M et al., 2020) (Chen Y et al., 2019) (Liu Y et al., 2020) (Khursheed R et al., 2020). Therefore, the exploration of new natural active ingredients with excellent pharmacological effects and long-term safety have become the research direction of new drugs for the treatment of T2DM.

*Cordyceps militaris* was known as medicinal and edible fungi with significant blood glucose lowering (Wang F et al., 2015) (Dong Y et al., 2014) (Gong X et al., 2021) (Hsu CH et al., 2008). It can be used as new resource food and raw material of health food. Compared with other medicinal fungi, few studies have been focused on the mechanism of action of *C. militaris* and its active components in the treatment of T2DM. *Cordyceps militaris* belongs to the entomopathogenic fungi, *lavicipitaceae* and *Ascomycotina*, which is widely used in medicines and health products. *Cordyceps militaris* has been shown to have antidiabetic effects and contains various active ingredients such as polysaccharide, cordycepin, adenosine and various trace elements (Dong Y et al., 2014). Among these active substances, cordycepin, a unique active ingredient of *C. militaris*, has been shown to decrease blood glucose and regulate dyslipidemia (Wang F et al., 2015), reduce inflammation (Gong X et al., 2021), relieve oxidative stress (Hsu CH et al., 2008), and promote immune regulation (Gong X et al., 2021), anti-tumor (Hsu CH et al., 2008), etc. It is reported that cordycepin can significantly regulate the intestinal microflora (An Y et al., 2018). However, the specific mechanism by which cordycepin and *C. militaris* extract improves T2DM is not yet fully understood.

Gut microbiota and metabolites are important factors in mediating the development of T2DM, an imbalance in the gut microbiota can affects glucose and lipid metabolism, thereby promoting the occurrence and development of metabolic diseases such as T2DM, non-alcoholic fatty liver disease (Zhou M et al., 2021), etc. Recently, a large number of edible fungi have been proved to alleviate T2DM by regulating the intestinal flora, leading us to explore whether the gut microbiota and gut barrier were involved in the beneficial effect of cordycepin and *C. militaris* extract on T2DM.

In general, microbiomics was used to analyze the structure of gut microbiota community, and metabolomics was used to analyze the metabolites of gut microbiota to explore the relationship between metabolites and disease. The research on intestinal flora mainly focuses on microbiome and metabolomics (Li L et al., 2021). To the best of our knowledge, few studies have combined gut microbiome and metabolomics to elucidate the effects of medicinal and edible fungi on T2DM, and none of them has been reported related to *C. militaris*. The purpose of this research is to evaluate the mechanism of cordycepin and *C. militaris* extract on alleviating the symptoms of T2DM from the perspectives of gut microbiome and metabolomics. This work has laid a theoretical foundation for the research on the treatment of T2DM with medicinal and edible fungi.

## 2 Materials and methods

### 2.1 Microorganism and materials

The anamorph strain JY20 of *C. militaris*, originally conserved in our lab, was confirmed by means of both morphological and molecular methods. Potato dextrose liquid medium was used as fermentation medium for 5 days. The mycelium was then inoculated to rice medium in glass jars and cultured in the dark at 22°C for 7 days, then at 22°C for 10 days, with a 10:14 h light/dark cycle for conversion of the fungi and forming stromata, with a temperature difference of more than 10°C between day and night for 28 days to form the mature fruiting body (Zhao X et al., 2019). The cultured stroma of *C. militaris* was lyophilized and ground through a 60-mesh sieve. The extraction of lyophilized fermentation product (60 mesh) was carried out three times, each time with 20-fold volume deionized water under ultrasound at 25°C for 30 min. The extract was centrifuged at 5,000 rpm for 15 min. The combined supernatants were evaporated at 55°C under reduced pressure, lyophilized, and then stored at −20°C for further analysis. Then, 1 g of lyophilized powder dissolved in ultrapure water, filtered through a 0.22 μm membrane to a final volume of 20 mL.

STZ (HPLC≥98%) (Solarbio Science and Technology company, cat. No. S8050), Metformin hydrochloride tablets (Glucophage^®^ 500 mg tablets) (Sino-American Shanghai Squibb Pharmaceutical company, cat. No. H20023371), Cordycepin (HPLC≥98%) (Yuanye Biotechnology company, cat. No. B20196), stored at dark and low temperature, and diluted to the desired concentration prior to use. High-fat feed (Xiaoshuyoutai Biotechnology company). Total cholesterol (TC), Triglyceride (TG), Low-density lipoprotein cholesterol (LDL-C), How-density lipoprotein cholesterol (HDL-C), Alanine aminotransferase (ALT), Superoxide dismutase (SOD), Catalase (CAT) kits (JianchengNanjing, China). The kits of Interleukin-6 (IL-6) and Tumor necrosis factor-α (TNF-α) (Boshen Biotechnology company, China).

### 2.2 Animal experiments

Seventy male Kunming mice (8 weeks old, 40 ± 2 g) were purchased from Vital River Laboratory Animal Technology company (Beijing, China) and housed in polypropylene cages (*n* = 5 mice/cage). Animals were housed at 22°C ± 2°C on a 12 h light/dark cycle and allowed free access to food and water. All methods and experimental protocols in the research process were approved by the Ethics Committee for Animal Research of School of Life Sciences, Shandong University (NO: SYDWLL-2021-29), and the protocols conformed to the U.S. Public Health Service Policy on Use of Laboratory Animals.

After the 7-day acclimation period, all mice were randomly divided into control group (ND, *n* = 10) and model group (T2DM, *n* = 60). The control group was fed with a normal diet, while the model group was fed with a 60% high-fat diet. After 4 weeks, all mice were fasted but had free access to water for 18 h. The model group mice were induced by intraperitoneal injection of STZ (70 mg/kg, dissolved in sodium citrate buffer, 0.1 mol/L, pH = 4.5) for 5 days. Meanwhile, the control group were injected with the same volume of citrate buffer. On the fifth day after injection, the level of 12 h fasting blood glucose (FBG) was measured in each group, and mice with FBG ≥11.1 mmol/L were considered as T2DM mice (Lu JM., 2016). Subsequently, all the T2DM mice were randomly divided into six groups (*n* = 10): model control group (HFD); metformin group (PC, 350 mg/kg); Cordycepin high-dose group (CCSH, 50 mg/kg); Cordycepin low-dose group (CCSL, 25 mg/kg); *C. militaris* extracts high-dose group (CEH, 1.5 g/kg); *C. militaris* extracts low-dose group (CEL, 1 g/kg). All mice were fed their respective diets until the end of the study period. Mice in the CCS groups, CE groups and PC group were gavaged for six consecutive weeks, while those in the ND and HFD groups were given the same volume of physiological saline (0.9%). The body weight of mice and FBG were measured every week. Fecal samples were aseptically collected at the fourth and sixth week of treatment. At the end of the experiment, the mice were fasted overnight, sacrificed under ether anesthesia. The blood samples were collected from the eyes and centrifuged (3000 r/min, 15 min) to obtain serum for biochemical analysis. All fecal and serum samples were cryopreserved at −80°C until analysis.

### 2.3 Metabolic parameters

Biochemical analysis of TC, TG, HDL-C, LDL-C, ALT and oxidative stress analysis of SOD, CAT and enzyme-linked immunosorbent assay of IL-6, TNF-α were executed by a microplate reader (Vlctor-x3, PerkinElmer, Waltham, MA, USA) according to protocols.

### 2.4 Gut microbiome

The special regions (16S V3V4) of 16S rRNA genes were sequenced to study the diversity of gut microbes. DNA was extracted from the fecal samples using a commercial DNA extraction kit (DNeasy PowerSoil Kit, German). DNA concentration and integrity were measured by a NanoDrop 2000 spectrophotometer (Thermo Fisher Scientific, Waltham, MA, United States) and agarose gel electrophoresis, respectively. Then DNA was sequenced using NovaSeq6000 platform (Illumina, California, United States) with a universal primer pair (343F: 5′-TACGGRAGGCAGCAG-3'; 798R: 5′-AGG​GTA​TCT​AAT​CCT-3′) by OE Biotech company (Shanghai, China). The raw data were processed using QIIME software (version 1.8.0). Then, clean reads were subjected to primer sequences removal and clustering to generate operational taxonomic units (OTUs) using VSEARCH software with 97% similarity cutoff. All representative reads were annotated and blasted against Silva database (Version 132) using RDP classifier (confidence threshold was 70%) (Wang Q et al., 2007). The alpha diversity and beta diversity were analyzed using QIIME software.

### 2.5 Untarget metabolomics

In this experiment, gas chromatography-mass spectrometry and liquid chromatography-mass spectrometry were used to study the metabolomics of fecal samples of mice after drug intervention for 6 weeks. In GC-MS, derivatized samples were analyzed on an Agilent 7890B gas chromatography system coupled to an Agilent 5977A MSD system (Agilent Technologies, CA, United States). Separation was performed on an Agilent DB-5MS fused silica capillary column (30 m × 0.25 mm × 0.25 μm). Mass spectrometry conditions were electron impact ionization (EI) source, ion source temperature 330°C, and transfer line temperature 280°C. The scanning mode is full scan, and the mass scanning range is m/z 50–500.

LC-MS was performed using a Dionex Ultimate 3000 RS UHPLC coupled with a Q-Exactive + quadrupole-orbitrap mass spectrometer equipped with a heated electrospray ionization (ESI) source (Thermo Fisher Scientific, Waltham, MA, USA) to analyze ESI positive Metabolic profiles in ion and negative ion mode. The column was a Waters ACQUITY UPLC HSS T3 (1.8 μm, 2.1 × 100 mm) in positive and negative modes.

### 2.6 Statistical analysis

The study of gut microbiome was evaluated by alpha diversity and beta diversity. The criteria for screening differential metabolites were VIP value greater than 1.0 and *p*-value less than 0.05. Metabolic pathway enrichment analysis of differential metabolites was performed based on the KEGG database. All statistical data were analyzed using SPSS software (version 25.0; SPSS, Chicago, IL, United States). GraphPad Prism (version 8.0) for creating all drawings. Data are presented as the means ± standard error of mean (SEM) values. Comparisons between multiple groups were evaluated using one-way ANOVA followed by LSD *post hoc* test. A *p*-value < 0.05 was considered to indicate statistical significance.

## 3 Results

### 3.1 Effects of CCS and CE on FBG and body weight of T2DM

**FIGURE 1:**
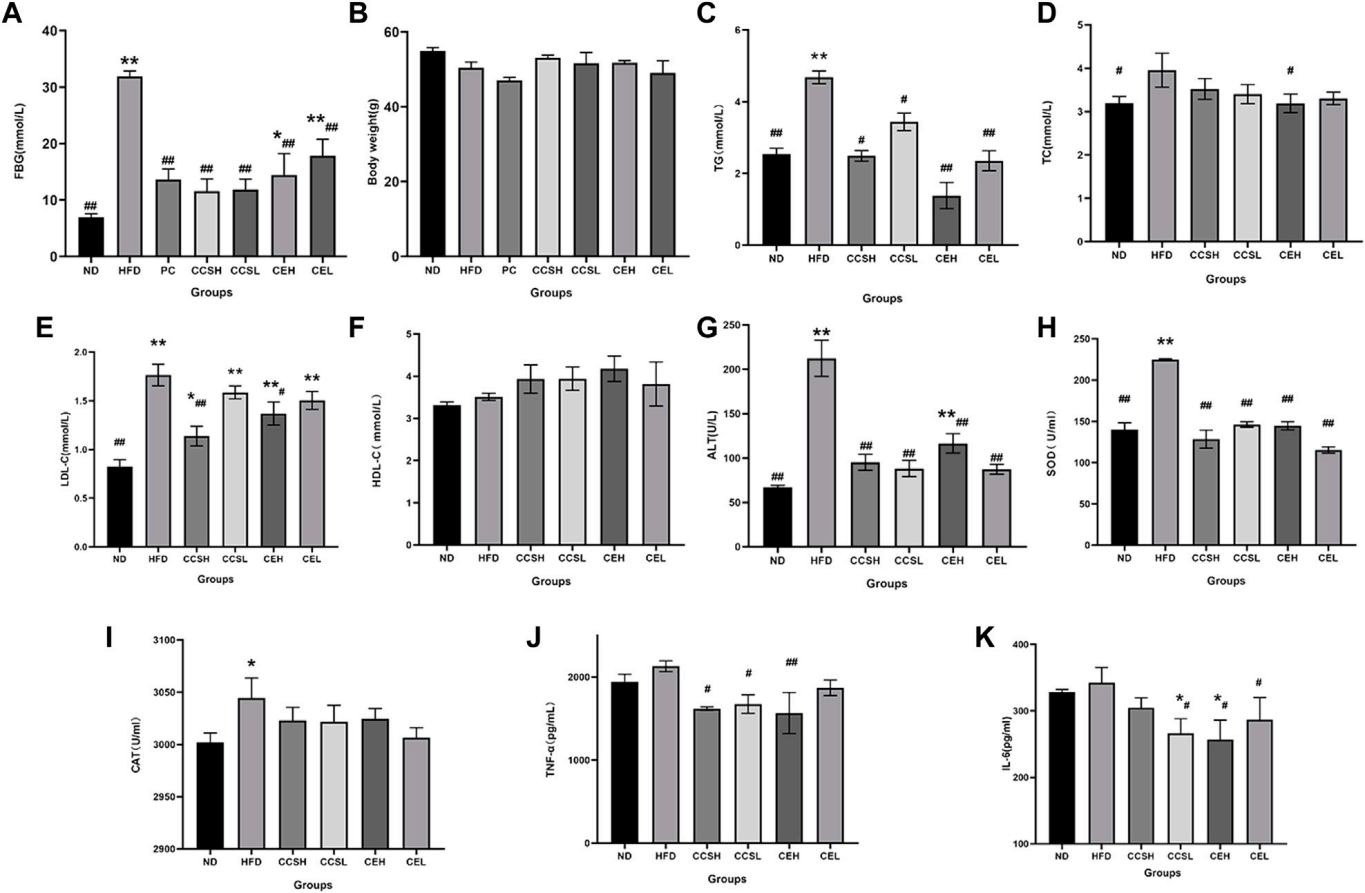
Effects of CCS and CE on physiological and biochemical parameters in T2DM. **(A)** FBG; **(B)** Body weight; **(C)** TG; **(D)** TC; **(E)** LDL-C; **(F)** HDL-C; **(G)** ALT; **(H)** SOD; **(I)** CAT; **(J)** TNF-α; **(K)** IL -6. Values are expressed as means ± SEM. Differences were assessed by ANOVA and denoted as follows: **p < 0.01, *p < 0.05 vs. ND (*n* = 10), ^##^ *p* < 0.01, ^#^ *p* < 0.05 vs. HFD (*n* = 10).

After 4 weeks of intragastric administration, compared to the first week, the level of fasting blood glucose was increased, and the HFD group showed obvious emaciation, uneven coat color and weight loss, urine output and water intake was elevated, as in previous studies (Zhang F et al., 2022). After 4 weeks of gavage, fasting blood glucose gain induced by HFD was lower when CCS and CE were administered (*p < 0.01*) (Figures 1A,B).

### 3.2 Effects of CCS and CE on metabolic parameters in T2DM

It was showed that compared with ND group, the levels of TC, TG and LDL-C in HFD group were significantly increased (*p* < 0.01), and HDL-C was significantly decreased, indicating that T2DM had symptoms of dyslipidemia. CCS and CE groups showed increased levels of TC, TG, LDL-C and decreased levels of HDL-C (Figures 1C–F). The expression of ALT, SOD, and CAT in the HFD group was increased compared to ND group (Figures 1G–I), indicating that the T2DM mice had liver damage and presented oxidative stress, while the intervention of CCS and CE significantly reduced the levels of oxidative factors (*p* < 0.01). To verify the effects of cordycepin and *C. militaris* extract on inflammatory factors in T2DM mice, enzyme-related immunosorbent assay was performed. The results showed that the levels of TNF-α and IL-6 in HFD group were higher than those in ND group, indicating a more obvious inflammatory response in T2DM mice. After CCS and CE treatment, the levels of TNF-α and IL-6 in treatment group were significantly lower than those in HFD group (Figures 1J,K).

### 3.3 CCS and CE modulate the community structure of gut microflora

**FIGURE 2:**
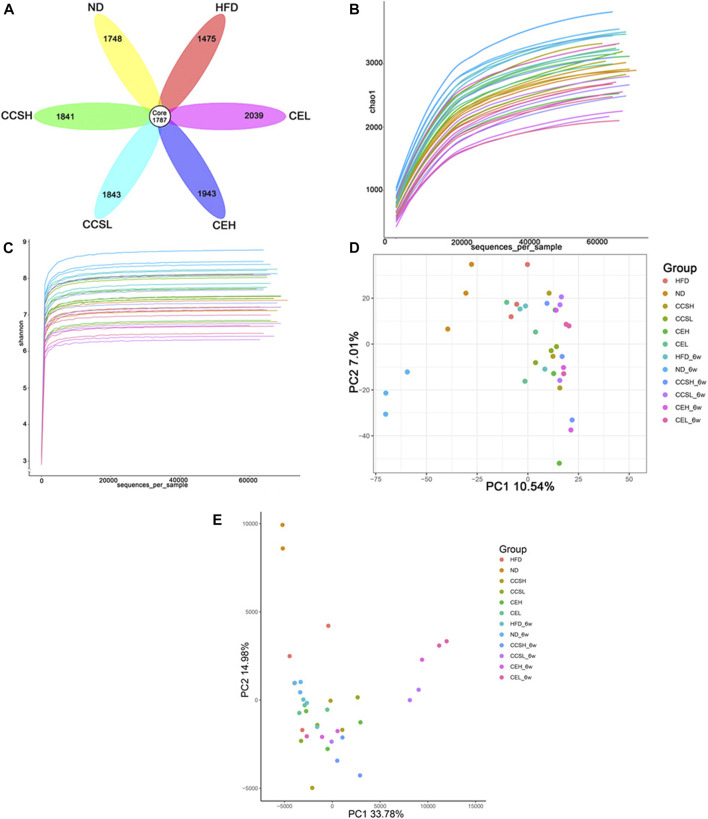
CCS and CE modulate the community structure of the gut microbiota. **(A)** OTUs petal images of each treatment group after 4 weeks of gavage. Core OTUs represent OTUs common to all groups, and numbers on petals represent OTUs unique to each group. **(B)** Sparse curve **(C)** Shannon index **(D)** PCA principal component analysis **(E)** Euclidean-based PcoA.

The species diversity of the samples was evaluated at the Operational Taxonomy Units (OTUs) level. At the 4st and 6st week of gavage, mice feces were taken for 16S rRNA gene sequencing. It is observed that 1787 OTUs are jointly owned by each group, while the OTUs unique to HFD group are less than those of the ND group, and the other treatment groups have more OTUs compared to controls, indicating that CCS and CE effectively suppressed HFD-induced intestinal microbial flora diversity reduce (Figure 2A).

The alpha and beta diversity indices were assessed for each group of gut microbiota (Figures 2B,C). Through the Chao1 index analysis and the displayed sparse curve, the trend shows that the sample selection is reasonable (Figure 2B). The Shannon-Weaver curve reflect species diversity for each sample as a sequencing function and the curve tends to be flat, indicating that the amount of sequencing data is large enough to reflect the vast majority of microbial species information in the sample (Figure 2C).

Principal component analysis (PCA) was performed by variance decomposition, and it can be seen that there was significant clustering among samples within the group, and the treatment group was significantly separated from the ND group (Figure 2D). After suffering from T2DM, some changes have occurred in the gut microbiota of mice. Similarly, principal co-ordinates analysis (PCoA) based on euclidean algorithm showed that the composition of gut microflora in each group had obvious aggregation. Multivariate analysis of the variance of the PCoA matrix scores revealed a statistically significant separation between the microflora of each group (Figure 2E).

### 3.4 CCS and CE improve gut microbiota composition in T2DM

**FIGURE 3:**
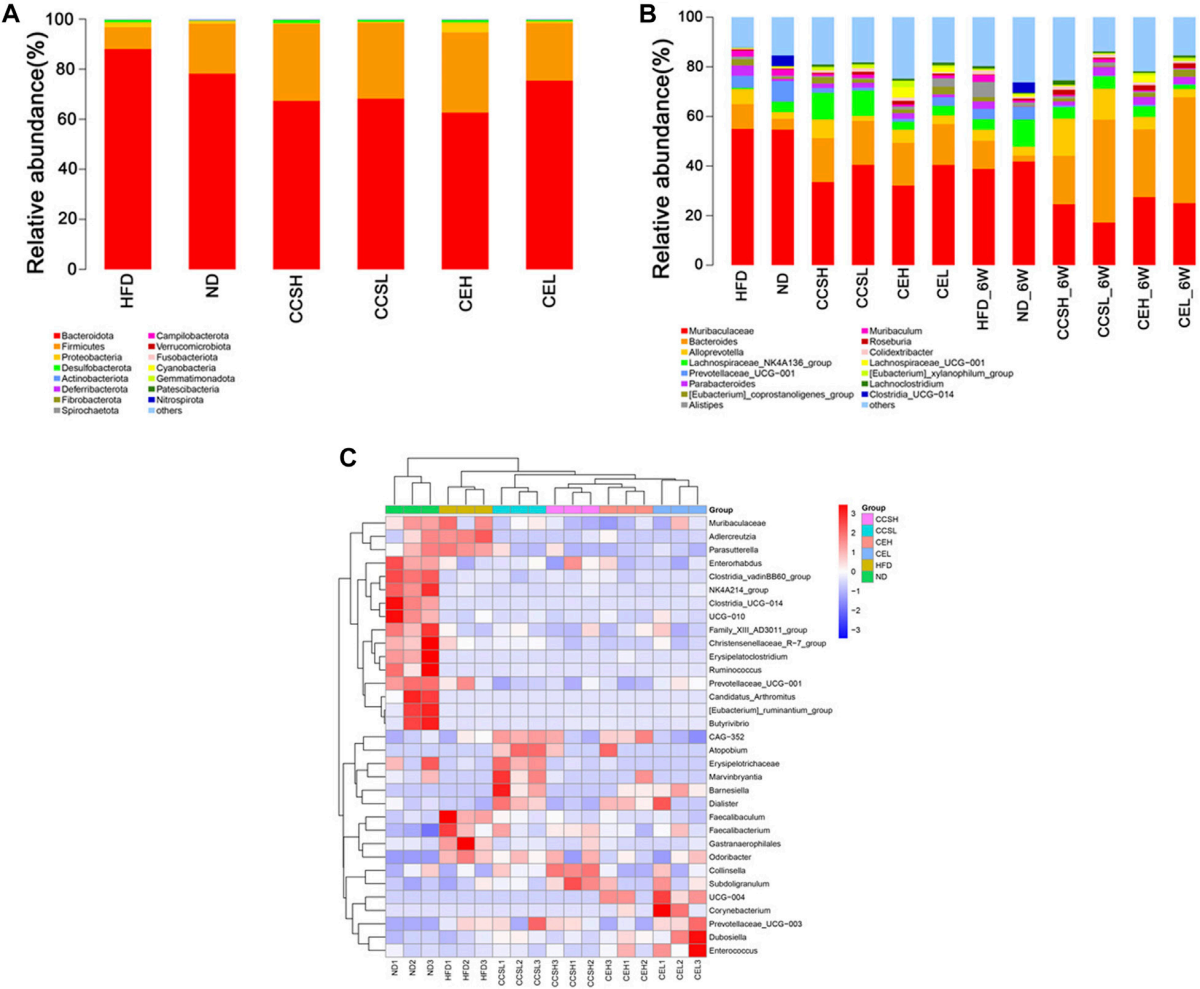
Effects of CCS and CE on the community structure of gut microflora in T2DM. **(A)** The distribution of community structure at the phylum level of each treatment at the fourth week after gavage group (Top 15); **(B)** Community structure distribution of each treatment group at the fourth and sixth week of gavage at the genus level (Top 15); **(C)** Heatmap analysis of the differential species of gut microbes at the genus level at the fourth week of gavage.

*Firmicutes* is a common indicator of gut microbiota balance, and bacteria of the phyla *Firmicutes* and *Bacteroidetes* were dominant in each group. The top 15 phyla of the relative abundance of intestinal microorganisms in each group after 4 weeks treatment is displayed in the form of a column chart. Remarkably, the relative abundance of *Firmicutes* increased in each treatment group compared with HFD group, and showed a dose-dependent (Figure 3A). We can see that the relative abundance (%) of *Firmicutes*, *Bacteroidetes* and the ratio of Firmicutes/Bacteroidetes in each group. Among them, F/B in ND group was 0.2558, HFD group was 0.0985, CCSH, CCSL, CEH and CEL group were 0.4551, 04432,0.5115 and 0.3039, respectively (Table 1). It was found that both CCS and CE improved the gut microbiota community structure in T2DM.

**TABLE 1:** Relative abundance of Firmicutes/Bacteroidetes among groups after 4 weeks treatment.

Major phyla	Relative abundance (%)					
	HFD	ND	CCSH	CCSL	CEH	CEL
Firmicutes	0.0867	0.2000	0.3065	0.3024	0.3205	0.2293
Bacteroidetes	0.8806	0.7820	0.6734	0.6824	0.6266	0.7545
Firmicutes/Bacteroidetes	0.0985	0.2558	0.4551	0.4432	0.5115	0.3039

In general, CCS and CE had a profound impact on the composition and abundance of the gut microbiome. From the 4st week to the 6st week of intervention, the relative abundance of *Bacteroides* increased, while that of Muribaculaceae decreased in CCS and CE groups. In addition, after 4 weeks of intervention, CCS and CE groups increased the relative abundance of *Colidextribacter* compared to controls; the relative abundance of *Eubacterium_xylanophilum_group* in each treatment group was higher than that in the ND group (Figure 3A). After 6 weeks of intervention, the relative abundance of *Alloprevotella* in treatment group except the CEL group was higher than that in ND group, especially in the cordycepin group, CCSH (0.1496), CCSL (0.1251); Compared with ND group, HFD significantly increased the relative abundance of *Muribaculum*, nevertheless, CCSH and CE treatments down this trend. *Roseburia* in the HFD group (0.0027) was lower than that in ND group (0.0081), while that in the CCS, CEH, and CEL groups were 0.0202, 0.0204, and 0.0199; Concomitantly, CE reduced the proportion of *Lachnoclostridium* (Figure 3B). In the heatmap of differential species at the four s t week of gavage, we can see that compared with the ND group, the relative abundance of some genera in the CCS and CE groups showed a downward trend, specifically, *Adlercreutzia*, *Prevotellaceae-UCG-001*. The relative abundance of *Odoribacter* in CCS and CE group declined significantly compared with that in HFD group. Overall, CCSL upregulated the relative abundance of *Atopobium*, *Erysipelotrichaceae*, *Marvinbryantia*, *Barnesiella*, *Dialister*, CCSH upregulated the relative abundance of *collinsella* and *Subdoligranulum*, Separately, CE could upregulate the relative abundance of *Corynebacterium*, *Prevotellaceae_UCG−003*, *Dubosiella*, and *Enterococcus* (Figure 3C).

### 3.5 Multivariate statistical analysis of metabolomics

**FIGURE 4:**
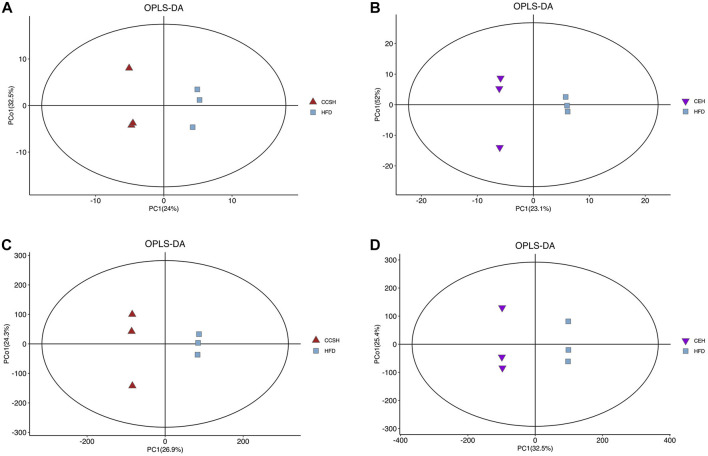
OPLS-DA analysis. **(A)** CCSH vs. HFD detected by GCMS; **(B)** CEH vs. HFD detected by GC-MS; **(C)** CCSH vs. HFD detected by LC-MS; **(D)** CEH vs. HFD detected by LC-MS.

GC-MS, LC-MS was used to detect metabolic information of intestinal contents in positive and negative ion modes. The position of the coordinate point represents the degree of dispersion of each sample in OPLS-DA. GC-MC detection results showed that the samples of CCSH group and HFD group were obviously separated (Figure 4A). CEH group and HFD group showed a separation trend in different quadrants (Figure 4B). Similarly, the comparison between CCSH and CE group and HFD group also showed a similar trend in the detection results of LC-MS (Figures 4C,D). Overall, the intestinal flora of the treatment group (CCSH and CEH) was significantly different from that of the model group (HFD).

### 3.6 Volcano plots of differential metabolites

We screened the differential metabolites based on the volcano maps, and the screening criteria were that the VIP value of the first principal component of the OPLS-DA model was greater than 1, and the *p*-value of the *t*-test was less than 0.05.

**FIGURE 5:**
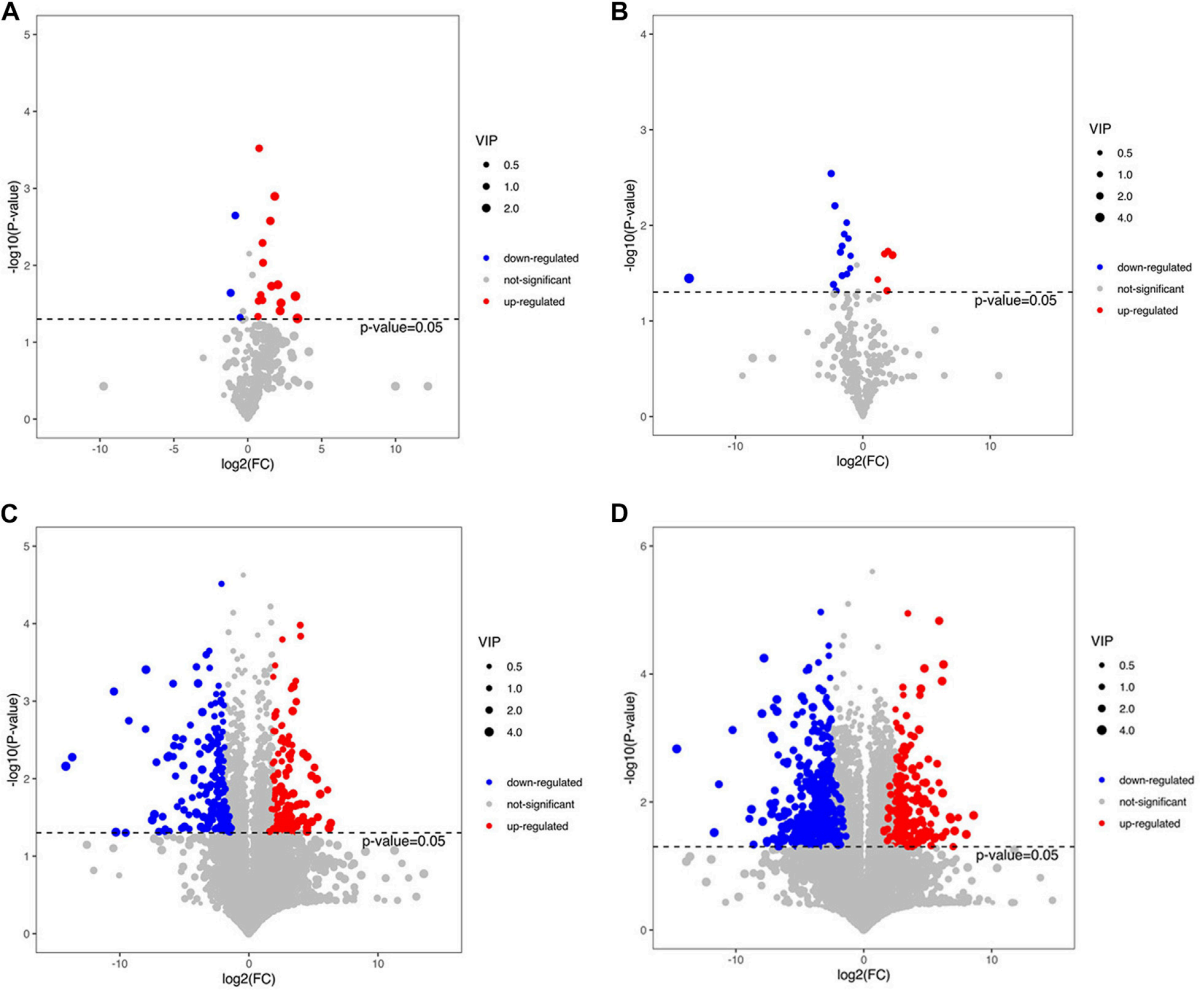
**(A)** Volcano plots of CCSH vs. HFD in GC-MS assay **(B)** CEH vs HFD in GC-MS assay. **(C)** Volcano plots of CCSH vs HFD in LC-MS assay. **(D)** Volcano plots of CEH vs HFD in LC-MS assay.

Compared with group HFD, 18 differential metabolites were detected in group CCSH by GC-MS detection, among which 15 were upregulated and three were downregulated. When group CEH was compared with HFD, 19 different metabolites were found, among which 5 were upregulated and 14 were downregulated (Figures 5A,B). A total of 363 differential metabolites were detected in group CCSH and group HFD by LC-MS, including positive and negative ion modes. Among them, 198 metabolites (78 upregulated and 120 downregulated) were significantly changed in positive ion mode. Then in negative ion mode, Significant changes were observed in 254 metabolites (89 upregulated and 76 downregulated). Similarly, a total of 699 differential metabolites were detected in the comparison between group CEH and HFD. Among them, 371 metabolites (122 upregulated and 249 downregulated) were significantly changed in positive ion mode, and 328 metabolites (121 upregulated and 207 downregulated) were significantly changed in negative ion mode (Figures 5C,D).

### 3.7 Clustering hierarchy of differential metabolites

**FIGURE 6:**
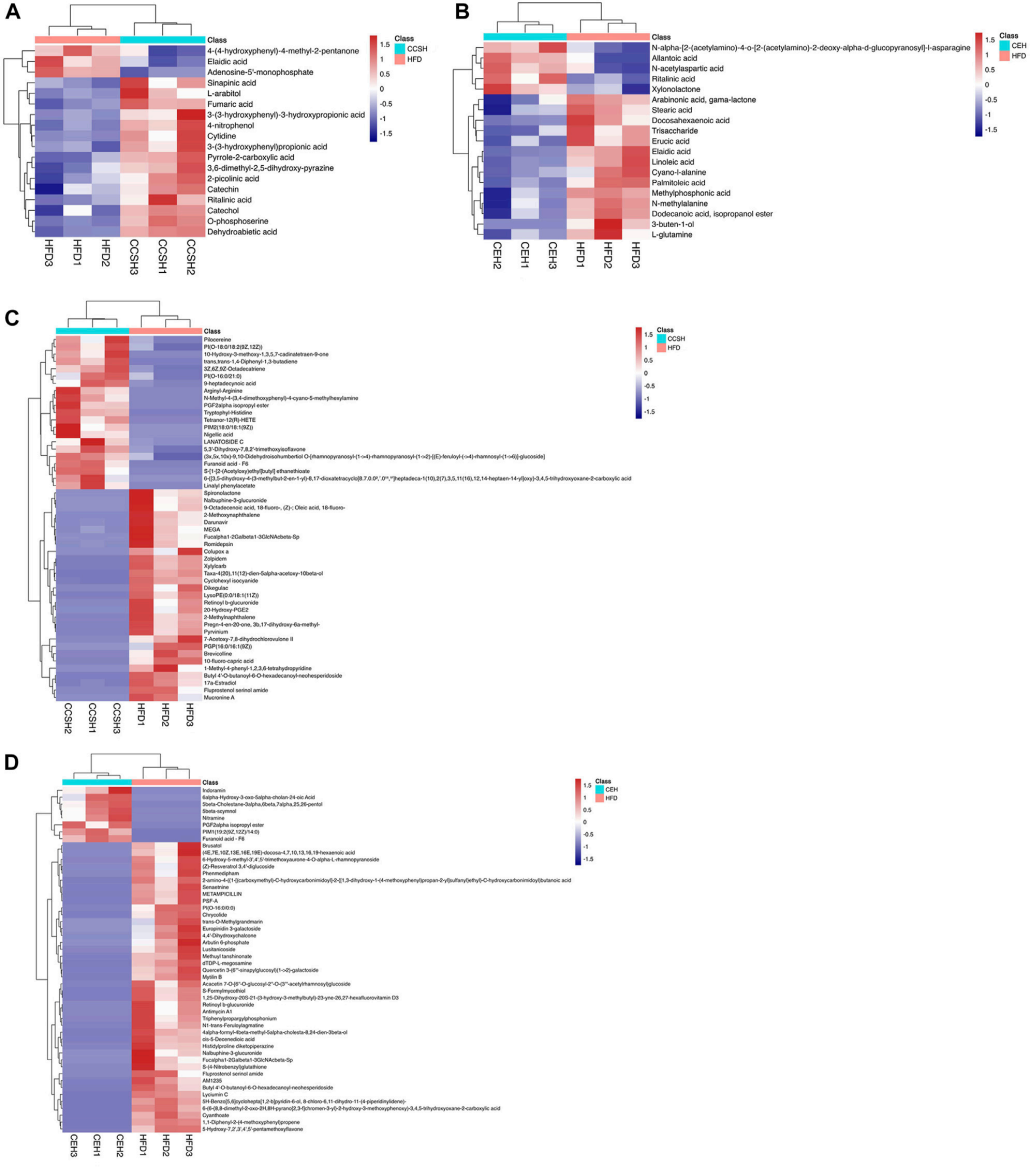
Heatmap of differential metabolites in fecal samples. **(A)** CCSH vs. HFD detected by GC-MS **(B)** CEH vs. HFD detected by GC-MS **(C)** CCSH vs HFD detected by LC-MS **(D)** CEH vs. HFD detected by LC-MS.

In order to more visually show the relationship between samples and the differences in metabolite expression between different samples, we performed systematic clustering of significantly different metabolite expression levels. Analysis of GC-MS results showed that compared with group HFD, some components included in group CCSH presented higher levels, such as phenyl propanoic acids, pyrimidine nucleosides, phenols, carboxylic acids and derivatives, prenol lipids, pyridines and derivatives, flavonoids (Figure 6A). Meanwhile, downregulated metabolites include purine nucleotides, fatty acyls. Similarly, carboxylic acids and derivatives were upregulated metabolites in group CEH compared with group HFD, and downregulated metabolites include fatty acyls, amino acids, organooxygen compounds (Figure 6B).

According to LC-MS detection, more altered metabolites were observed. Compared with group HFD, the metabolites upregulated in CCSH group included glycerophospholipids, isoflavonoids, fatty acyls, carboxylic acids and derivatives, prenol lipids, thiocarboxylic acids and derivatives. Several metabolites were downregulated such as steroids and steroid derivatives, benzene and substituted derivatives, glycerophospholipids, pyrroles, fatty acyls, naphthalenes, prenol lipids, benzofurans, azoles, carboxylic acids and derivatives (Figure 6C). In addition, glycerophospholipids, sterol lipids, fatty acyls belonging to the group CEH, occurred at higher levels than group HFD. Meanwhile, downregulated metabolites in group CEH include isoflavonoids, carboxylic acids and derivatives, benzofurans, cinnamic acids and derivatives, prenol lipids, organooxygen compounds, polyketides, sterol Lipids, coumarins and derivatives, fatty acyls, glycerophospholipids, stilbenes (Figure 6D).

### 3.8 Metabolic pathways analysis in KEGG

**FIGURE 7:**
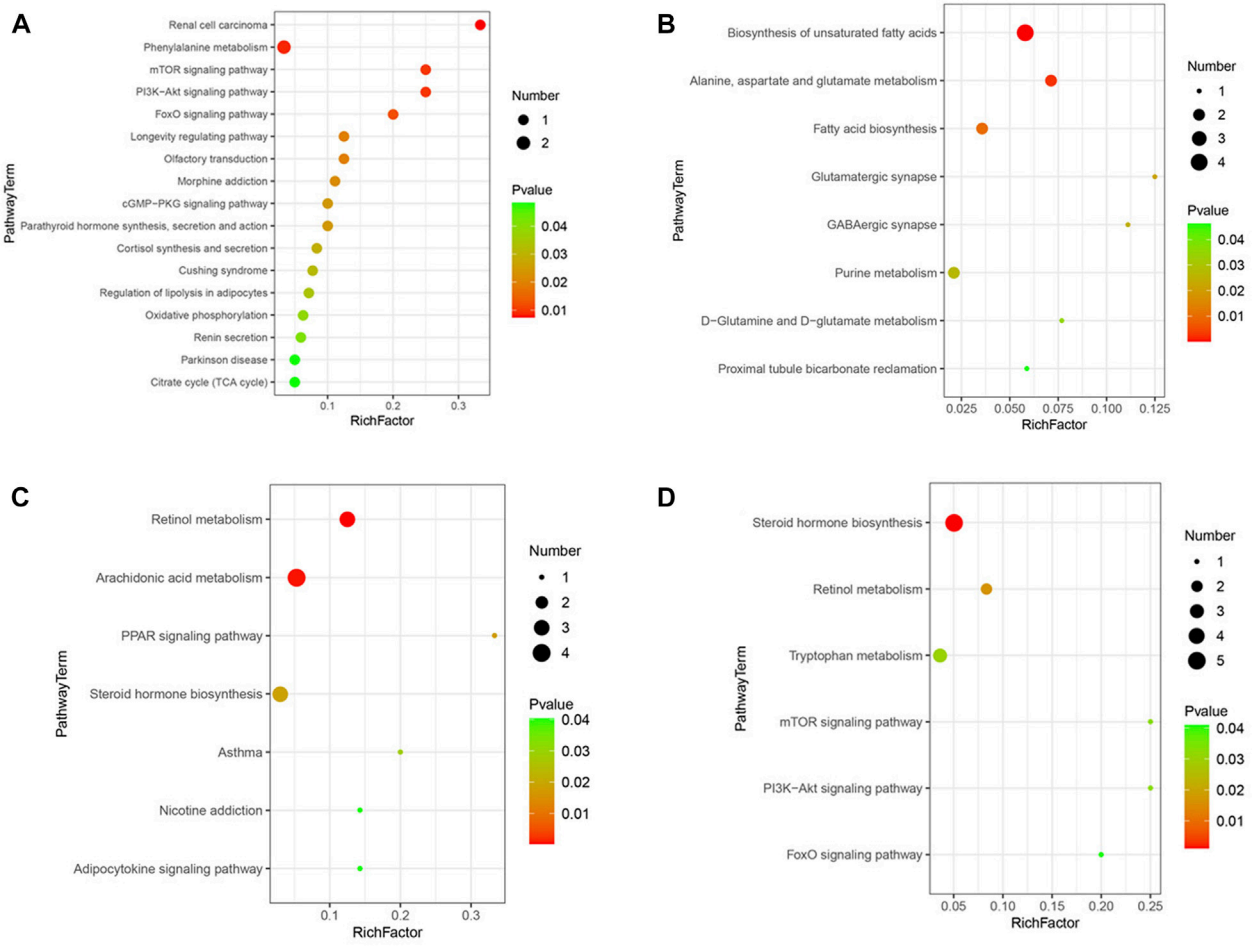
Metabolic pathway bubble diagram. **(A)** CCSH vs HFD metabolic pathways in stool samples detected by GC-MS (*p* < 0.05) **(B)** CEH vs HFD detected by GC-MS **(C)** CCSH vs HFD detected by LC-MS **(D)** CEH vs HFD detected by LC-MS (*p* < 0.05).

In the comparison of CCSH and HFD by GC-MS, after the introduction of KEGG, metabolites of CCSH can screen out some metabolic pathways under the condition of *p < 0.05* (Figure 7A). Such as mTOR signaling pathway, PI3K-Akt signaling pathway, FOXO signaling pathway, cGMP-PKG signaling pathway, citrate cycle (TCA cycle) pathway. Similarly, metabolic pathways were screened in CCSH and HFD by LC-MS detection method (Figure 7B). Specifically, including: retinol metabolism pathway, arachidonic acid metabolism pathway, PPAR signaling pathway, adipocytokine signaling pathway.

Results shown that, some metabolic pathways were screened by GC-MS in the comparison of CEH with HFD. Specifically, it includes D-glutamine and D-glutamate metabolism, proximal tubule bicarbonate reclamation, etc. (Figure 7C). The results of LC-MS detection showed that some metabolic pathways were screened out by comparison between CEH and HFD. Specifically, it includes tryptophan metabolism pathway, PI3K-Akt signaling pathway, FOXO signaling pathway (Figure 7D).

## 4 Discussion

The purpose of this study was to explore the potential mechanisms of cordycepin and its aqueous extract in alleviating the symptoms of T2DM. Results showed that high-fat diet led to weight loss, loss of luster of hair and increased the level of FBG. The level of FBG in the metformin group, CCS and CE group was significantly lower than that in the HFD group (*p < 0.01*), suggesting that cordycepin and *C. militaris* extract had potential blood glucose lowering effects in T2DM.

Compared with ND group, the levels of TC, TG, and LDL-C in HFD group were significantly increased (*p < 0.01*), and HDL-C was significantly decreased, which indicated that T2DM would lead to dyslipidemia. The levels of TC, TG and LDL-C in the treatment group were lower than those in HFD group, which proves that CCS and CE could regulate the lipid metabolism of T2DM. The content of cordycepin in CCSL was 122 times that of CEL by HPLC. Nevertheless, the downregulation of TG level by CE was more significant, which shows that there were other potential lipid-regulating active substances in *C. militaris* extract.

HFD group had higher levels of proinflammatory cytokines and excessive oxidative stress Compared to controls, Cordycepin and *C. militaris* extract may exert beneficial effects by reducing inflammatory factors and attenuating oxidative stress. The levels of ALT and SOD in T2DM were significantly increased, which was alleviated by CCS and CE (*p < 0.01*), indicating that cordycepin and *C. militaris* extract had potential effects on the antioxidant capacity and liver protection of mice. Compared with the ND group, the level of IL-6 in HFD group increased, while CCS and CE significantly downregulated these inflammatory factors, suggesting that CCS and CE had potential effects in regulating the inflammatory response.

Recent studies have highlighted that many ingredients from natural plants exhibited bifunction in model organisms by mediating hormesis (Jiang et al., 2020a). Hormesis is a biphasic dose-response relationship characterized by low dose stimulation and high dose inhibition, and is typically represented as a J-shaped or inverted U-shaped curve (Sun H et al., 2020). Extracts of many herbs, either individually or in combination, can trigger hormetic phenomena in different models *in vitro*, including animal and human cells.

In the comparison of high and low doses of CCS group, there were no significant differences in biochemical indexes such as CAT, ALT and IL-6, etc. It is explained that at low doses, it may already be in the second half of the “J” curve in the dose-response curve model, approaching the flat part. Low doses can already reduce the index, and the difference in effect between high and low doses is not significant, so there is no obvious dose dependence. In the comparison of high and low doses in the CE group, the biochemical indexes ALT and CAT had a more obvious therapeutic effect at low doses than at high doses, and IL-6 was also more effective at low doses in the CCS group. We speculate that the cause may be similar to the biphasic dose-effect, such as an inverted U-shaped curve: within a certain range, low concentrations have a stimulative effect, while high concentrations relatively diminish this stimulative effect. This suggests that dosing studies for cordycepin and water extracts are important and need to be further explored.

The dose of Chinese herbal medicine has been widely concerned. The active substances in Coptis chinensis have a biphasic dose effect on the regulation of detoxifying enzymes GST and CarE, which shows an increasing trend at low dose and a decreasing trend at high dose (Jiang et al., 2020b). Our study suggests that *C. militaris* as a dietary supplement, the effects of different doses still need attention.

By sequencing the 16S rRNA gene, we can intuitively see the changes of intestinal microflora structure of CCS and CE groups, so as to explore its regulatory effect on intestinal microflora of T2DM. In this study, the rationality of sample selection was determined by evaluating the *a*-diversity and *ß*-diversity indices of intestinal flora in each group. In PcoA based on Euclidean, it can be seen that the samples in each group have obvious aggregation. After 6 weeks of intragastric treatment, the intestinal flora structure of CCS and CE groups tends to approach ND, which indicates that CCS and CE can improve the intestinal flora disorder of T2DM. From the OTU Venn diagram after 4 weeks of gavage treatment, it can be seen that T2DM reduced the diversity of microbiota, and both CCS and CE increased the diversity of intestinal microbiota (Figure 2A). After 4 weeks of gavage, at the phylum level, compared with ND group, the relative abundance of *Firmicutes* in the HFD group decreased significantly, while that in CCS and CE groups increased, showed a dose-dependent trend, combined with the ratio of Firmicutes/Bacteroidetes, indicating that both CCS and CE can ameliorate the composition of gut microbiota in T2DM.

The community structure distribution map at the genus level showed that T2DM changed the gut microbial community structure of mice, and the relative abundances of *Alistipes*, *Muribaculum*, and *Lachnoclostridium* in HFD group were higher than those in ND group. Compared to controls, *Parabacteroides* elevated in the CCS and CE groups. Studies have shown that *Parabacteroides distasonis* is one of the core floras of the human body, NAFLD, DM and other disease states are significantly negatively correlated, and may play a positive regulatory role in glucose and lipid metabolism (Wang K et al., 2019). The relative abundances of *Eubacterium_xylanophilum_group* and *Colidextribacter* in CCS and CE groups were higher than those in ND group. *Eubacterium_xylanophilum_group* could interfere with the catabolism of Branched-Chain Amino Acid (BCAA) to alleviate high-fat induction the body weight of obese mice (Zhang L et al., 2020); *Colidextribacter* was proved to be an inosine-producing bacterium, which could change the intestinal microbial structure and improve LPS-induced acute liver injury and inflammation by regulating the TLR4/NF-κB signaling pathway, indicating that CCS, CE has a certain positive effect on preventing liver damage and reducing obesity symptoms (Guo W et al., 2021). After 6 weeks of gavage, the relative abundance of *Roseburia* in CCSH, CEH, CEL groups was significantly higher than that in HFD group. *Roseburia* is a butyric acid-producing bacteria that degrades dietary fiber xylan in the intestinal tract, and butyric acid secreted by gut microbiota will Promote postprandial insulin secretion (Leth ML et al., 2018), suggesting that CCS and CE may reduce blood sugar of T2DM by influencing intestinal microflora. *Alloprevotella* belongs to short-chain fatty acid-producing bacteria and anti-inflammatory bacteria, the amplitude of *Alloprevotella* in CCS was significantly greater than that in HFD group, indicating that CCS has a positive effect on anti-inflammatory and short-chain fatty acid production (Sanna S et al., 2019); The treatment of CE inhibited the HFD-induced increase in the relative abundance of *Lachnoclastic*, metagenomics showed that *Lachnoclastic* could be used as a marker for the diagnosis of colorectal adenoma and colon cancer, indicating that the active components in CE could effectively protect and prevent colorectal cancer (Liang JQ et al., 2020). Notably, *Clostridia_UCG-014* showed a high relative abundance only in ND group, and has been studied relatively rarely to date (Figure 4B).

Metabolomics studies of fecal samples show that the differential metabolites of CCSH and HFD are rich in some pathways related to DM, in particular, peroxisome proliferator-activated receptor (PPAR) pathway, arachidonic acid pathway, PI3K/Akt pathway, mTOR signaling pathway, FOXO signaling pathway, oxidative phosphorylation chemical pathway, TCA cycle pathway, adipokine signaling pathway. These pathways may play an important role in the regulation of blood glucose and dyslipidemia by CCS.

PPARs are member of the nuclear receptor transcription factor superfamily that regulate the expression of target genes. Three isoforms were found in different species: PPARα, PPARβ/δ, and PPARγ. PPARs are key regulators of glucose homeostasis and lipid metabolism, and also important targets for the development of modern anti-diabetic drugs to improve insulin sensitivity and blood glucose level by regulating target genes (Jiang et al., 2020a). PPARs regulate gene transcription by initially activating by binding to ligand fatty acids and their derivatives, forming heterodimers with retinoid X receptor, and then binding to DNA sites of specific sequences to induce target gene activation (Chan and Wells, 2009), thereby regulating lipid metabolism, adipocyte differentiation, glycogenesis, ubiquitination, and inflammation (Gross B et al., 2017). In the comparison of CCSH and HFD, PPAR signaling pathway was enriched. According to KEGG, we speculate that CCSH could regulate lipid metabolism, reduce inflammatory response and insulin resistance through PI3K/Akt/PPAR signaling pathway.

Arachidonic acid (AA) is the precursor of many bioactive substances, which can significantly prevent early insulin resistance induced by a high-fat diet (Wang B et al., 2021). Three different enzyme systems in the metabolic pathway of AA, cyclooxygenase, lipoxygenase and cytochrome P450, can produce important unsaturated fatty acids (Carboneau BA et al., 2017). Prostaglandin (PG) produced by AA metabolism plays an important role in the dysfunction of *ß*-cells and insulin secretion. Among them, PGI2 can promote pancreatic *ß*-cells to secrete insulin and prevent STZ-induced hyperglycemia in mice by maintaining the mass of pancreatic *ß*-cell and plasma insulin level (Li X et al., 2021). This approach may be one of the important ways for CCSH to improve diabetes by regulating endocrine and other physiological processes.

The PI3K/Akt pathway is an upstream regulation pathway of mTOR, which is involved in the uptake and utilization of glucose of cells and plays an important role in cell growth and metabolism. In addition, the PI3K/Akt/mTOR pathway plays a key role in glucose homeostasis, Therefore, CCSH can regulate glucose metabolism through this pathway. FOXO is a fork head transcription factor that involved in glucose metabolism and regulating gluconeogenic genes including PEPCK and G6Pase. After dephosphorylation, FOXO is in an activated state, which leads to gluconeogenesis (Agus et al., 2018). It is regulated by upstream PI3K/Akt pathway, and loses its activity after phosphorylation, which inhibits the expression of gluconeogenesis genes, thus lowering blood sugar. Overall, we speculate that CCSH is involved in the regulation of glycolysis and gluconeogenesis through PI3K/Akt/FOXO/PEPCK pathway.

Similarly, the PI3K/Akt/mTOR pathway, the PI3K/Akt/FOXO/PEPCK pathway still existed in CEH group. In addition, through metabolic pathway enrichment analysis, we found that some amino acid metabolic pathways were changed after CEH treatment, specifically, including the metabolic pathway of tryptophan, alanine-aspartate-glutamate metabolic pathway and D-Glutamine and D-glutamate metabolic pathways. Tryptophan has been proved to be converted to serotonin (Karamitri and Jockers, 2019), which in turn is converted to melatonin. Studies have shown that melatonin is related to insulin resistance, which can improve insulin resistance and participate in regulating glucose homeostasis. It can be seen that amino acid metabolism plays a role in the process of CEH regulating blood sugar. Therefore, the mechanism of CEH regulating blood sugar may be related to the metabolism of amino acids.

The differential metabolites of CCSH and HFD included phenols, fatty acids, phenylacetones, pyrimidine nucleosides, carboxylic acids and their derivatives, pyridines and their derivatives. Particularly, Catechin of phenylacetone compound can reduce lipid accumulation and increase the number of islets *ß* cells in T2DM (Wang TJ et al., 2011), which has a potential role in the treatment of T2DM. Among the differential metabolites of CEH and HFD, there are alcohols, amino acids, fatty acids, etc. Studies have shown that some branched-chain amino acids, aromatic amino acids and their derivatives, and fatty acids are highly correlated with the development of diabetes (Ahola-Olli AV et al., 2019). Erucic acid is a fatty acid that has a significant positive correlation with lipid, lipoprotein synthesis, and elevated plasma glucose (Ghosh A, 2007). High content of erucic acid can easily lead to cardiomyopathy and fat deposition. The erucic acid level in CE group was significantly lower compared with that in HFD group, which indicated that CE could improve blood glucose metabolism and blood lipid metabolism.

According to the metabolomics analysis results of different fecal samples, it is obvious that our mice produce many different metabolites through the interaction between intestinal microbes and the host, which are associated with many metabolic pathways, mainly focusing on PI3K/Akt/PPAR, PI3K/Akt/mTOR, PI3K/Akt/FOXO/PEPCK pathway, tryptophan metabolism, alanine diurnal acid-glutamic acid metabolism pathway, D-glutamine and D-glutamic acid metabolism pathway, unsaturated fatty acid synthesis pathway, TCA cycle pathway. It can be speculated that cordycepin and *C. militaris* extract may affect insulin resistance, blood sugar level, amino acid metabolism, fatty acid metabolism, anti-inflammatory ability and energy supply in T2DM through these pathways, thus alleviating the symptoms of T2DM.

## 5 Conclusion

In conclusion, our study suggests that the mechanism of *C. militaris* intervention on T2DM may be to improve the abundance of Firmicutes/Bacteroidetes, promote the growth of beneficial bacteria, and regulate the intestinal flora structure and enhance the metabolites and metabolic pathways associated with T2DM alterations. Thereby improving physiological and biochemical indicators and relieving the symptoms of type 2 diabetes. In addition, this study combined with molecular biology experiments, 16S rDNA sequencing and metabolomics detection, for the first time to explore the potential mechanism of CCS and CE regulating intestinal flora and metabolites to improve T2DM. Furthermore, our results suggest that *C. militaris*, as a kind of edible fungus, can alleviate T2DM in mice, and provide a scientific foundation for improving human health by using *C. militaris*.

## Data Availability

The datasets presented in this study can be found in online repositories. The names of the repository/repositories and accession number(s) can be found below: https://www.ncbi.nlm.nih.gov/, PRJNA832098 https://figshare.com/s/0df8d0cc07e297824e7d.

## Abbreviations

AA, arachidonic acid; ALT, alanine aminotransferase; BCAA, branched-chain amino acid; CAT, catalase; CE, *cordyceps militaris* extracts; CCS, cordycepin; DM, diabetes mellitus; ESI, electrospray ionization; FBG, fasting blood-glucose; GC-MS, gas chromatograph- mass spectrometer; HDL-C, high-density lipoprotein cholesterol; HPLC, high performance liquid chromatography; IL-6, interleukin-6; LC-MS, liquid chromatography- mass spectrometry; LDL-C, low-density lipoprotein cholesterol; OPLS-DA, orthogonal projections to latent structures; OTUs, operational taxonomy units; PCA, principal component analysis; PCoA, principal coordinates analysis; PBS, phosphate buffered saline; PG, prostaglandin; PPARs, peroxisome proliferators-activated receptors; SOD, superoxide dismutase; STZ, streptozocin; T2DM, type 2 diabetes mellitus; TC, total cholesterol; TCA, tricarboxylic acid; TG, triglyceride; TNF-α, tumor necrosis factor-α.
